# Accuracy of standard clinical 3T prostate MRI for pelvic lymph node staging: *Comparison to*^*68*^*Ga-PSMA PET-CT*

**DOI:** 10.1038/s41598-019-46386-3

**Published:** 2019-07-24

**Authors:** Sebastian Meißner, Jan-Carlo Janssen, Vikas Prasad, Gerd Diederichs, Bernd Hamm, Winfried Brenner, Marcus R. Makowski

**Affiliations:** 1 Department of Radiology, Charité, Charitéplatz 1, 10117 Berlin, Germany; 2 Department of Nuclear Medicine, Charité, Charitéplatz 1, 10117 Berlin, Germany

**Keywords:** Magnetic resonance imaging, Positron-emission tomography, Cancer imaging, Prostate

## Abstract

The aim was to assess the performance of prostate 3T MRI for pelvic lymph node (LN) staging in prostate cancer (PCa), in comparison to ^68^Gallium-prostate specific membrane antigen PET-CT (^68^Ga-PSMA PET-CT) as reference standard for LN detection. 130 patients with PCa underwent non-contrast-enhanced multiparametric prostate 3T MRI and ^68^Ga-PSMA-PET-CT within 180 days at our institution. Overall, 187 LN metastases (n = 43 patients) detected by ^68^Ga-PSMA-PET-CT were characterized by calculating maximum standardized uptake value (SUVmax), area, diameter and anatomical location including iliac, obturator, presacral and inguinal region. MRI achieved an overall sensitivity, specificity, positive and negative predictive value of 81.6% (CI 71.1–88.9%), 98.6% (CI 97.6–99.2%), 73.5% (CI 52.1–87.6%) and 99.5% (CI 98.8–99.8%), respectively. On a region-based analysis, detection rates differed non-significantly (ps > 0.12) in the anatomical regions. On a size-dependent analysis, detection of LN > 10 mm did not differ significantly (ps > 0.09) from LN ≤ 10 mm. In comparison to single T1 sequence evaluation, additional use of the T2 weighted sequences did not improve the overall performance significantly (p > 0.05). 3T prostate MRI represented an accurate tool for the detection of LN compared to ^68^Ga-PSMA-PET-CT. Especially for LN metastases smaller than 10 mm, MRI was less accurate compared to ^68^Ga-PSMA-PET-CT.

## Introduction

Prostate cancer (PCa) is the second leading cause of death in men and the most common tumor in men in the western hemisphere^1^. Therapeutic strategies are highly dependent on the assessment of the lymph node (LN) status, since it affects the therapy regimen and overall prognosis in newly diagnosed PCa^2^. Prior to radical prostatectomy, current guidelines recommend CT or MRI for local and LN staging as well as bone scintigraphy for osseous metastasis for patients with intermediate to high risk of recurrence according to D’Amico’s classification^2,3^. Even though choline PET is associated with certain limitations regarding the sensitivity and specificity for LN detection, it has been established as part of the clinical routine in certain centers^4^. Regarding MRI, especially T1 and T2 sequences are known to provide excellent anatomical information to demarcate structures and are commonly used for evaluation of local tumor infiltrations for instance in the seminal vesicles as well as local LN evaluation^5–12^. With the introduction of 3T scanners into the clinical setting, higher signal to noise ratios could be realized and a more reliable detection of LN metastases could be achieved^5,13–15^.

Recently, PET was put back into focus for PCa staging, when novel probes targeting cell-surface receptors of PCa cells were introduced^16–18^. The prostate membrane antigen (PSMA) is a highly expressed protein located at the cell surface of PCa cells and the use of ^68^Gallium radiolabeled PSMA inhibitor Glu-urea-Lys(Ahx)-HBED-CC showed promising results in detection of primary and recurrent disease^19,20^.

The aim of this study was to assess the performance of clinical routine prostate 3T MRI for pelvic LN staging in PCa, compared to ^68^Gallium-prostate specific membrane antigen PET-CT (^68^Ga-PSMA PET-CT) as reference standard for LN detection.

## Results

### Detection of lymph node metastases in MRI

Of 130 patients, 43 patients harbored 187 LN metastases in ^68^Ga-PSMA PET-CT with a mean area, size ratio and SUVmax of 0.9 ± 1.1 cm^2^ (range 0.1–9.2 cm^2^), 0.7 ± 0.2 (range 0.3–1.5) and 10.2 ± 11.5 (range 0.7–75.5). MRI detected 146 LN with a mean area of 1.0 ± 1.2 cm^2^ (range 0.1–9.1 cm^2^) and a size-ratio of 0.8 ± 0.5 (range 0.3–3.5). MRI achieved an overall sensitivity, specificity, PPV and NPV of 81.6% (CI 71.1–88.9%) 98.6% (CI 97.6–99.2%), 73.5% (CI 52.1–87.6%) and 99.5% (CI 98.8–99.8%). 41 false negative LN had a mean area, size ratio and SUVmax of 0.4 ± 0.3 cm^2^ (range 0.1–1.8 cm^2^), 0.7 ± 0.1 (range 0.4–1.0) and 6.1 ± 3.8 (range 0.7–18.9) in ^68^Ga-PSMA-PET-CT. 1130 true negative LN were measured in ^68^Ga-PSMA-PET-CT with a mean area of 0.5 ± 0.3 cm^2^ (range 0.1–3.5 cm^2^) and a size-ratio of 0.7 ± 0.2 (range 0.2–4.5). An overall of 35 false positive LN were detected in MRI with a mean area of 1.1 ± 0.7 cm^2^ (range 0.2–3.0 cm^2^) and a size-ratio of 0.7 ± 0.2 (range 0.4–1.0). Characteristics for all 1317 LN in ^68^Ga-PSMA-PET-CT are presented in Table 1 and descriptive statistics of the LN found in MRI are displayed in Table 2.

**Table 1:** Characteristics of all investigated lymph nodes in ^68^Gallium-Prostate specific membrane antigen PET-CT.

	Count	Area ± SD [cm^2^] (mean)	LAdm ± SD [mm] (mean)	SAdm ± SD [mm] (mean)	Size ratio ± SD [mm] (mean)
All LN	1317	0.5 ± 0.5	9.2 ± 3.8	6.2 ± 2.4	0.7 ± 0.2
Iliac left	243	0.4 ± 0.6	8.3 ± 3.7	5.4 ± 2.4	0.7 ± 0.2
Iliac right	210	0.5 ± 0.8	9.2 ± 4.4	6.0 ± 2.9	0.7 ± 0.2
Obturator left	104	0.5 ± 0.6	8.2 ± 4.3	5.8 ± 3.1	0.7 ± 0.2
Obturator right	106	0.5 ± 0.6	8.9 ± 4.9	5.9 ± 2.6	0.7 ± 0.2
Presacral	26	0.3 ± 0.4	8.1 ± 2.7	6.0 ± 2.1	0.7 ± 0.1
Inguinal	628	0.6 ± 0.4	9.9 ± 3.2	6.7 ± 2.0	0.7 ± 0.2

This table presents the main characteristics of all lymph nodes (LN) depicted in ^68^Gallium-Prostate specific membrane antigen PET-CT including all benign and malignant LN. Count, area, long-axis diameter, short-axis diameter and size-ratio of the LN are presented in the columns. The size ratio is described as quotient of short-axis diameter divided through long-axis diameter. The rows show all LN together and fielded in the six defined anatomical regions iliac left and right, obturator left and right, presacral and inguinal region. Data are given in means and standard deviations.

Abbreviations: LN = Lymph nodes, LAdm = Long-axis diameter, SAdm = Short-axis diameter, SD = Standard deviation.

**Table 2:** Characteristics of all PET-positive lymph nodes depicted in MRI using T1 combined with T2 and DWI-sequences.

	Area ± SD [cm^2^] (mean)	LAdm ± SD [mm] (mean)	SAdm ± SD [mm] (mean)	Size-ratio ± SD (mean)
All LN	1.0 ± 1.2	11.4 ± 6.3	8.4 ± 4.0	0.8 ± 0.5
Iliac left	1.0 ± 1.8	11.7 ± 7.3	8.7 ± 5.2	0.7 ± 0.2
Iliac right	1.3 ± 1.3	14.2 ± 6.3	9.8 ± 4.4	0.7 ± 0.2
Obturator left	1.0 ± 1.1	10.3 ± 6.6	8.3 ± 4.4	1.0 ± 0.7
Obturator right	0.8 ± 1.0	11.1 ± 5.5	7.7 ± 2.6	0.9 ± 0.6
Presacral	0.5 ± 0.4	8.6 ± 3.0	6.7 ± 2.6	0.8 ± 0.2
Inguinal	1.0 ± 1.2	12.5 ± 7.1	8.5 ± 4.8	0.7 ± 0.2

This table presents the main characteristics of all malignant lymph nodes (LN) depicted in ^68^Gallium-Prostate specific membrane antigen PET-CT and MRI. Area, long-axis diameter, short-axis diameter and size ratio of the LN are presented in the columns. The size ratio is described as quotient of short-axis diameter divided through long-axis diameter. The rows show all LN together and fielded in the six defined anatomical regions iliac left and right, obturator left and right, presacral and inguinal region. Data are given in means and standard deviations.

Abbreviations: LN = Lymph nodes, LAdm = Long-axis diameter, SAdm = Short-axis diameter, SD = Standard deviation.

We thus were interested in possible impacts, that may have altered the results. The time between the two measures PET-CT and MRI varied between 0 to 180 days but the diagnostical accuracy did not suffer significantly from longer time intervals between the scans (χ²(2) = 1.2, p = 0.55). The impact of patients with multiple lesions to diagnostic accuracy was not significant (χ²(2) = 2.1,p = 0.36) and was deducted in all results given.

### Detection of lymph node metastases in MRI dependent on anatomical regions

In the analysis dependent on anatomical regions the sensitivities differed between 66.8% and 100% while specificities ranged between 97.1% and 100%. The highest detection rate was presented in the inguinal region with a 100% (CI 0–100%) followed by the obturator right and left region with 88.8% (CI 75.0–95.5%) and 84.9% (CI 62.8–95.0%). Presacral LN were detected with a sensitivity was 82.0% (CI 50.0–95.4%). The lowest sensitivity was presented in the iliac region with 80.7% (CI 60.0–92.1%) for the left and 66.8% (CI 46.6–82.2%) for the right region. Next, we evaluated possible differences in MRI diagnostical accuracy in the different anatomical regions. The six regions had a significant additional impact on diagnostical accuracy beyond the MRI judgments (χ²(10) = 18.7, p < 0.05). However, the source of this impact could not be pinpointed, as all single interaction effects were non-significant in logistic regression (ps > 0.12). Differences in the diagnostical accuracy were calculated relative to the iliac left region, which was arbitrary and was non-significant as can be seen at the overlapping CI of the sensitivities and specificities throughout the regions. Please refer to Table 3 for further details.

**Table 3:** Overall and region-based detection rate using combined T1 + T2 + DWI sequence MRI evaluation.

T1 + T2	N	Sensitivity + 95% CI	Specificity + 95% CI	PPV + 95 95% CI	NPV + 95% CI
All LN	146/187	81.6% (71.1–88.9%)	98.6% (97.6–99.2%)	73.5% (52.1–87.6%)	99.5% (98.8–99.8%)
Iliac left	23/31	80.7% (60.0–92.1%)	98.5% (95.8–99.4%)	52.5% (18.4–84.4%)	99.6% (98.0–99.9%)
Iliac right	30/47	66.8% (46.6–82.2%)	97.2% (93.7–98.8%)	72.5% (37.8–92.0%)	98.8% (95.0–99.7%)
Obturator left	28/33	84.9% (62.8–95.0%)	98.6% (95.4–99.6%)	85.1% (39.4–98.0%)	98.3% (87.1–99.8%)
Obturator right	44/52	88.8% (75.0–95.5%)	97.1% (92.7–98.9%)	91.8% (66.2–98.5%)	96.9% (84.3–99.4%)
Presacral	13/16	82.0% (50.0–95.4%)	100% (100–100%)	99.8% (0–100%)	95.9% (0–100%)
Inguinal	8/8	100% (0–100%)	99.2% (0–100%)	28.5% (5.3–73.8%)	100% (100–100%)

This table summarizes the region-based analysis of the lymph node metastases in MRI using T1 combined with T2 and DWI sequence evaluation. The columns present number, sensitivities, specificities, positive and negative predictive value including 95% confidence interval. The rows are fielded in the six defined anatomical regions iliac left and right, obturator left and right, presacral and inguinal region.

Abbreviations: LN = Lymph nodes, PPV = positive predictive value, NPV = Negative predictive value, CI = 95% Confidence interval.

### Assessment of histopathological data compared to MRI sensitivity

To assess a possible impact of histopathologic data to the readings, an interaction test was performed in this study. The diagnostical accuracy of MRI was significantly influenced by the results of the biopsy (χ²(1) = 4.8, p < 0.05). Sensitivity increases with greater Gleason scores from biopsy, i.e., p = 0.21 for a Gleason score of 6 and p = 0.97 for a score of 10. Gleason scores at the time of biopsy ranged from 6 to 10 with a mean of 7.8. Due to missing final Gleason scores after prostatectomy in most patients, no reliable statements could be made apart from biopsy.

### Impact of the additional T2 and DWI sequences in lymph node staging

**Figure 1:**
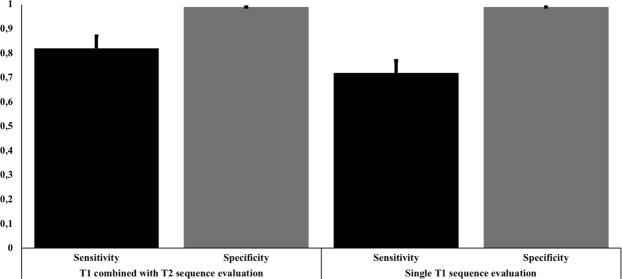
Visualisation of the detection rates of lymph node metastases according to the used MRI sequences. This bar chart presents the accuracy of the detection for lymph node (LN) metastases in T1 combined with T2 sequence evaluation versus single T1 sequence evaluation showing the overall sensitivities and specificities.

Since LN detection in MRI is commonly related to T1 sequence evaluation, we assessed the performance of T1 plus T2 and DWI compared to single T1 sequence evaluation. 130 LN were detected in the single T1 evaluation presenting an overall sensitivity, specificity, PPV and NPV of 71.8% (CI 58.2–82.3%), 99.0% (CI 98.3–99.5%), 72.2% (CI 47.9–88.0%) and 99.5% (CI 98.5–99.8%). Like T1 + T2, single T1 sequence evaluation displayed substantial diagnostical accuracy. Mean area was 1.0 ± 1.2 cm^2^ (range 0.1–9.1 cm^2^) and a mean size ratio of 0.8 ± 0.2 (range 0.3–1.) in MRI. False negative LN had a mean area, size ratio and SUVmax of 0.5 ± 0.3 cm^2^ (range 0.1–1.8 cm^2^), 0.7 ± 0.2 (range 0.3–1.5) and 6.4 ± 4.0 (range 0.7–18.9) in ^68^Ga-PSMA-PET-CT. False positive LN had a mean area of 1.2 ± 0.7 cm^2^ (range 0.3–3.0 cm^2^) and a size ratio of 0.7 ± 0.2 (range 0.4–1.0) in MRI. The sensitivities in single T1 sequence evaluation differed between 65.0% and 100% while specificities ranged between 97.7% and 100%. The highest detection rate was presented in the inguinal region with a 100% (CI 0–100%) followed by the presacral region with 82.3% (48.8–95.8%). LN in the obturator left region were detected with a sensitivity of 75.9% (CI 50.0–91.0%) followed by iliac left with 73.1% (CI 48.9–88.5%) and the obturator right region with 67.5% (CI 47.2–83.0%). The lowest sensitivity was seen in the iliac right region with 65.0% (CI 43.2–81.9%). Although the sensitivity from T1 alone was lower than combined with T2 + DWI, this difference was not significant, as can be seen from the overlapping CIs between single T1 versus T1 combined with T2 and DWI sequence evaluation. Please refer to Table 4 and Fig. 1 for all details.

**Table 4:** Overall and region-based detection rate using single T1 sequence MRI evaluation.

Single T1	N	Sensitivity + 95% CI	Specificity + 95% CI	PPV + 95 95% CI	NPV + 95% CI
All LN	130/187	71.8% (58.2–82.3%)	99.0% (98.3–99.5%)	72.2% (47.9–88.0%)	99.5% (98.5–99.8%)
Iliac left	21/31	73.1% (48.9–88.5%)	99.0% (97.1–99.6%)	53.9% (19.5–83.1%)	99.6% (98.1–99.9%)
Iliac right	28/47	65.0% (43.2–81.9%)	98.0% (95.2–99.2%)	71.7% (38.2–92.4%)	98.7% (98.7–99.7%)
Obturator left	24/33	75.9% (50.0–91.0%)	99.7% (98.3–99.8%)	98.4% (86.0–99.6%)	98.2% (85.9–99.6%)
Obturator right	36/52	67.5% (47.2–83.0%)	97.8% (95.0–99.0%)	86.2% (65.0–97.1%)	93.5% (86.6–98.9%)
Presacral	13/16	82.3% (48.8–95.8%)	100% (100–100%)	99.9% (0–100%)	97.0% (0–100%)
Inguinal	8/8	100% (0–100%)	99.3% (0–100%)	28.5% (18.4–36.6%)	100% (100–100%)

This table summarizes the region-based analysis of the lymph node metastases in MRI using single T1 sequence evaluation. The columns present number, sensitivities, specificities, positive and negative predictive value including 95% confidence interval. The rows are fielded in the six defined anatomical regions iliac left and right, obturator left and right, presacral and inguinal region.

Abbreviations: LN = Lymph nodes, N = number of positive LN in MRI versus PET-CT, PPV = positive predictive value, NPV = Negative predictive value, CI = 95% Confidence interval.

## Discussion

**Figure 2:**
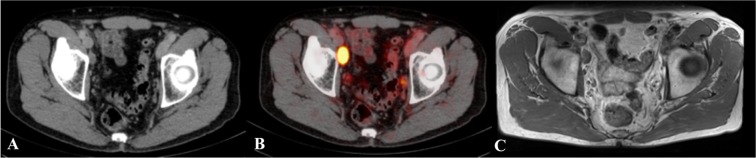
Example of a lymph node which is directly detectable on T1 MRI and ^68^Gallium-Prostate specific membrane antigen PET-CT. This figure shows a lymph node (LN) which is directly detectable on T1 MRI and ^68^Gallium-Prostate specific membrane antigen PET-CT. This large suspect LN is located at the right external iliac artery. The LN is visualized in corresponding axial plane slices using CT, ^68^Gallium-Prostate specific membrane antigen PET-CT and T1 sequence MRI presented left to right. (**A**) Lymph node depicted in CT, (**B**) Lymph node depicted in ^68^Gallium-Prostate specific membrane antigen PET-CT; (**C**) Lymph node depicted in the T1 sequence of MRI.

**Figure 3:**
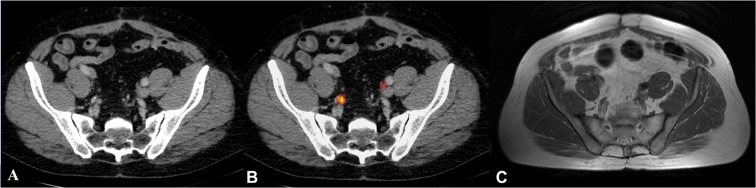
Example of a lymph node which is challenging to detect on T1 MRI compared to the ^68^Gallium-Prostate specific membrane antigen PET-CT. This figure presents an example of a lymph node close to the iliac bifurcation. The right and left external and internal iliac bifurcations are visualized in corresponding axial plane slices using CT, ^68^Gallium-Prostate specific membrane antigen PET-CT and T1 sequence MRI presented left to right. (**A**) Lymph node depicted in CT, (**B**) Lymph node depicted in ^68^Gallium-Prostate specific membrane antigen PET-CT; (**C**) Lymph node depicted in the T1 sequence of MRI.

This study demonstrated that high resolution 3 T prostate MRI represents an accurate tool for the detection of LN metastases. Especially for LN metastases smaller than 10 mm, MRI was less accurate compared to ^68^Ga-PSMA-PET-CT. In the region-based analysis, the performance of MRI was not significantly (p > 0.05) different throughout the anatomical regions. The highest detection rate for MRI was achieved for the inguinal, obturator and presacral region, while the lowest sensitivities were achieved in the iliac regions. This may be owed to the challenging anatomical conditions and flow artifacts resulting from these vessels. Detection rates were higher in areas where LN can be better delineated from surrounding structures. Figures 2 and 3 present examples of direct and challenging detection of LN in PET-CT and MRI.

**Figure 4:**
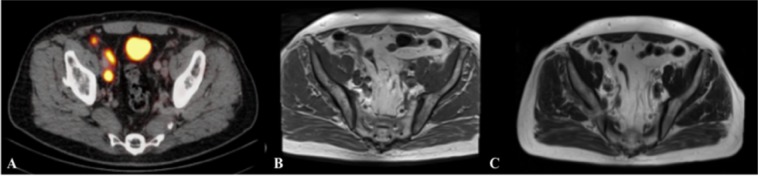
Lymph node evaluation depicted in ^68^Gallium-Prostate specific membrane antigen PET-CT and multiparametric MRI. This figure shows an example of lymph node (LN) detection using multiparametric MRI. The lymph node is visualized in corresponding axial plane slices using ^68^Gallium-Prostate specific membrane antigen PET-CT, T1 and T2 sequences of MRI presented left to right. The additional use of the T2 weighted sequence improved the detection of a LN in risk to be missed. This suspect LN is located at the right external iliac artery and is challenging to depict in the T1 sequence. (**A**) Lymph node depicted in ^68^Gallium-Prostate specific membrane antigen PET-CT, (**B**) Lymph node depicted in the T1 sequence in MRI; (**C**) Lymph node depicted in the T2 sequence in MRI.

In comparison to single T1 sequence evaluation, T1 combined with T2 and DWI sequence evaluation showed no statistically significant (p > 0.05) higher overall sensitivity and in the region-based analysis, underlining the relevance of sole T1 sequence evaluation. An example of additional T2 sequence evaluation is presented in Fig. 4.

On a size-based analysis, LN ≤ 10 mm in the iliac, obturator and presacral region were frequently missed, whereas LN ≤ 10 mm were detected more reliable in all anatomical regions but no statistically significant (p > 0.05) lower detection rate was seen for LN ≤ 10 mm compared to LN > 10 mm. Similar non-significant (p > 0.05) effects were found for single T1 sequence evaluation showing no significant additional impact of T2 evaluation based on LN size. For additional details please refer to the supplementary dataset.

^68^Ga-PSMA-HBEDD-CC is an inhibitor of the glutamate carboxypeptidase II, labelled with ^68^Gallium^21^. PSMA is a cell-surface transmembrane protein found in the prostate, brain, lacrimal and salivary glands, tumor neovasculature, tubules of the kidney and intestine^22^. It is a 110 kDa highly glycolysated peptidase and belongs to a family of zinc-dependent exopeptidases with glutamate carboxypeptidase activity^22,23^. It is highly active in prostatic intraepithelial neoplasia and metastatic PCa^22^. As of today, only few numbers of studies assessed the performance of ^68^Ga-PSMA PET-CT for LN staging in PCa patients and the diagnostic accuracy was tested regularly against histopathology as standard reference. Recently, Hamed *et al*. presented a prospective study with 106 out of 165 patients presenting with local recurrence and extraprostatic metastases, in which sensitivity, specificity and accuracy of ^68^Ga-PSMA was 99.0%, 100% and 98.8%, respectively, compared to histopathology^24^. In 2016, a study was published by Herlemann *et al*. on 34 patients undergoing a ^68^Ga-PSMA PET-CT prior to pelvic lymph node dissection (PLND) reporting an overall sensitivity, specificity, NPV and PPV of 84.0%, 82.0%, 84.0% and 82.0%, respectively^25^. Subsequent studies also demonstrated favorable detection rates for ^68^Ga-PSMA PET-CT in comparison to histopathology^26,27^. These results underline the value of this imaging technique, while PLND is an invasive procedure associated with perioperative risks such as lymphedema and venous thromboembolism^28^. Another major limitation of histopathological assessment is the sampling error^8^. Skip metastases near the common and internal iliac vessels can be missed due to the limited exploration in the surgery^8^.

Histopathological evaluation of LN is mainly dependent on morphologic criteria such as enlarged diameter or rounded LN or an increased LN volume^29^. Another input is set through the extranodal extension of LN metastases which is defined as a perforation of the LN capsule resulting in an expansion into extranodal tissues^30^. In 1998 Cheng *et al*. published a study including 269 patients, with LN metastases, presenting a significant correlation between nodal cancer volume and Gleason score recommending, that the diameter of the largest LN should be evaluated as prognostic factor of progression to distant metastasis rather than the number of LN^30^.In a follow-up study in 2000, Cheng *et al*. investigated the distant metastasis free and cancer-specific 5 year survival showing that extranodal extension was not significantly (p > 0.05) associated with distant metastasis free and cancer-specific 5 year survival in contrast to nodal cancer volume which was significantly (p > 0.05) associated with poorer 5 year survival^31^. A meta-analysis published in 2017 by Luchini *et al*. investigated the risk of recurrence through extranodal extension resulting in an elevated risk for patients with extranodal extension in PLND to develop a biochemical recurrence or distant metastasis^32^.

Today, MRI of the prostate is a clinical routine and highly sensitive imaging procedure for staging of patients and detection of extracapsular and seminal vesicle infiltration due to its excellent anatomical resolution^11,12^. The performance for LN staging is still considered to be challenging^12^. A meta-analysis of Hövels *et al*. included 24 studies with a mean sensitivity and specificity of 39.0% and 82.0% with ranges of sensitivities and specificities of 6.0–83.0% and 65.0–99.0%, respectively for LN detection by MRI prior to PLND while magnetic field strength however remained unknown for all investigated studies^5^. Sensitivity, specificity, PPV and NPV of 71.4%, 94.7%, 62.5% and 96.4%, respectively, were presented in a study by Kim *et al*. in 2010 for non-contrast enhanced T1 and T2 sequences of 1.5T MRI using surface coils for LN staging in comparison to histopathology^6^. In 2017, Gupta *et al*. reported sensitivity, specificity, PPV and NPV of 25.9%, 98.6%, 70.0% and 91.4%, respectively for LN detection using non contrast enhanced 1.5T MRI^9^.

Since area, LAdm, SAdm and size ratio are major signs for malignancy in MRI, high resolution 3T MRI showed an improved detection rate of small LN ≤ 10 mm in our study. Interestingly, a generally lower threshold for malignancy (LAdm of <10 mm) presented a better performance in comparison to histopathology than a higher threshold in a previous study, which is comparable to our findings^5^. In 2016, Barchetti *et al*. compared the performance of 1.5T MRI with non-contrast enhanced T1 plus T2 sequences in comparison to ^18^F-Choline PET-CT exams in 152 patients with biochemical recurrence reporting a sensitivity, specificity, PPV and NPV of 98.0%, 99.0%, 97.0% and 98.0%, respectively^10^. This good performance of 1.5T MRI has to be seen in the context of the chosen reference standard. Tulsyan *et al*. examined the usefulness of ^68^Ga-PSMA PET-CT in 36 patients with a biopsy proven PCa with a minimum Gleason score of 8 and PSA blood levels >20 ng/ml for LN staging in comparison to non-contrast enhanced 1.5T MRI^11^. 29 out of 36 patients presented LN in ^68^Ga-PSMA PET-CT in contrast to 20 MRI positive patients, which resulted in a concordance of 72% between both modalities without sensitivities or specificities given in the manuscript^11^.

Regarding the use of 3T MRI, Zattoni *et al*. published a study on recurrent PCa after failure of primary radiation therapy using histopathology as reference standard for LN detection by contrast enhanced 3T MRI applying an endorectal coil. They reported a sensitivity and specificity of 60.0% and 85.7%, respectively, for an unknown number of LN^15^. A further study from Zhang *et al*. compared contrast enhanced multiparametric MRI at 3T and ^68^Ga-PSMA PET-CT against histopathology for a cohort of 42 patients prior undergoing radical prostatectomy with PLND and found to be equal regarding the diagnostic accuracy^14^. A total of 51 LN out of 621 resected LN were defined malignant in histopathology and sensitivities, specificities, PPV and NPV of 96.1%, 99.5%, 94.2% and 99.7% for MRI and 96.1%, 99.7%, 96.1% and 99.7% for ^68^Ga-PSMA PET-CT were reported^14^. In our study ^68^Ga-PSMA-PET-CT was set as reference standard with an overall sensitivity, specificity, PPV and NPV of 81.6%, 98.6%, 73.5% and 99.5% for MRI.

In contrast to our study, Zhang *et al*. did not present LN detection rates according to anatomical regions while showing a higher overall detection rates with comparable results in the size-based analysis compared to our study. This may be due to the rather small cohort of 42 patients and the elevated mean PSA blood level with 52.3 ng/ml in Zhang *et al*. compared to 15.8 ng/ml in our study. Furthermore, the delay between PET-CT, MRI and surgery remained unknown. Moreover, the used malignancy criteria for MRI in Zhang *et al*. were SAdm > 10 mm, a rounded LN with a SAdm > 8 mm, increased contrast enhancement or a diffusion restriction in DWI and ADC map^14^. Our study chose a stricter malignancy criteria with a LAdm excess of 10 mm or a rounded LN defined through the quotient of SAdm divided through LAdm was present. In contrast to Zhang *et al*., the investigated MRI sequences in this study did not include the examination of DCE sequences and no use of contrast agent in our study, since gadolinium is associated with certain risks including nephrogenic systemic fibrosis especially in patients with chronic kidney disease^33^.

In summary, higher PSA blood levels may indicate more advanced tumor stages, the unknown delay of the procedures, the additional use of DCE sequences and contrast agent may have resulted in the slightly higher detection rates in Zhang *et al*. compared to our study. The comparable results of our study underline the validity of ^68^Ga-PSMA-PET-CT as reference standard compared to histopathology.

Limitations are the retrospective character of this study and the relatively small number of patients involved. No histopathological confirmation of the metastases seen in ^68^Ga-PSMA PET-CT was performed. A delay of up to 180 days between both imaging techniques might have influenced the size of the LN, but because PCa is a slow growing cancer entity, this time delay was considered of minor relevance. The performance of MRI detection was shortened through challenging localization near big vessels, and therefore interobserver variability cannot be excluded. Pitfalls of physiological tracer uptake were limited through parallel evaluation in CT. Size variations or necrosis of LN may have biased the examination as well. Given that size is a determining factor of malignancy in MRI, the size-based analysis could be biased leading to better sensitivities for LN > 10 mm compared to LN ≤ 10 mm. To limit this effect, the examination was done by two experienced readers and a size ratio was included to detect small but malignant LN based on a previous study in T1 sequences^29^.

In conclusion, high resolution 3T prostate MRI represents an accurate tool for the detection of LN metastases. The detection rates of MRI were lower for metastases in complex anatomical regions, compared to ^68^Ga-PSMA PET-CT. Especially for LN metastases smaller than 10 mm, MRI was less accurate compared to ^68^Ga-PSMA-PET-CT. The authors suggest that ^68^Ga-PSMA-PET-CT should be used for primary lymph node staging and for patients with biochemical recurrence.

## Methods

### Study population

The institutional ethics review board of the Department of Radiology at the Charité Universitätsmedizin Berlin, Germany approved this retrospective study and it was performed in accordance to current guidelines. The local database was screened for patients, who received a ^68^Ga-PSMA PET-CT and a 3 Tesla prostate MRI within 180 days. 1170 patients received a ^68^Ga-PSMA PET-CT between October 2013 and May 2018. No MRI or external MRI was available in the local database in 855 cases. 185 cases exceeded the maximum time interval of 180 days. The remaining cohort consisted of 130 patients. All Patients had histological proven prostate cancer with mean age of 72.1 ± 7.5 years (range 51–89 years) and received a ^68^Ga-PSMA PET-CT and a 3T MRI within 72.7 ± 48.4 days (range 0–180 days). The mean delay between diagnosis and the first examined scan (PET-CT or MRI) in this study was 1.7 ± 3.4 years (range 0–17 years) with a median of 0 years. When multiple studies were present, the scans with the shortest delay were used in this investigation. All other examinations were not used as part of the reading. Patients in this study did not receive any surgery, radiotherapy, change in the regime of chemotherapy or change of hormonal treatment within the delay between both modalities. There was no artificial delay of treatment in the patients investigated in this study. Prostate specific antigen (PSA) blood levels were 15.8 ± 23.6 ng/ml (range 0.2–196 ng/ml) collected within 26.5 ± 43.0 days (range 0–193 days) to the ^68^Ga-PSMA PET-CT and a median core needle biopsy Gleason score of 8 (range 6–10) was reported. Patients characteristics are presented in Table 5.

**Table 5:** Basic characteristics of study collective.

	mean	SD	median	range
Age (years)	72.1	7.5	73	51–89
Days ^68^GA-PSMA-PET to MRI	72.7	48.4	63	0–180
Days PSA to ^68^GA-PSMA-PET	26.5	43	0	0–193
PSA (ng/ml)	15.8	23.6	9.7	0.2–196
T-Stage	2.5	0.7	2.5	1–4
Prostate volume (ml)	68.8	36.0	68	10–188
Gleason score biopsy	7, 9	1.0	8	6–10
Gleason score after surgery	7.7	0.9	7	7–9

The basic characteristics of the prostate cancer patients who received a ^68^Gallium-Prostate specific membrane antigen (^68^Ga-PSMA) PET-CT and a multiparametric MRI within 180 days investigated in this study are presented in this table. This included the age of the patients, the delay between both imaging modalities, the prostate specific antigen blood level, the delay towards the ^68^Ga-PSMA PET-CT, prostate volumes, the Gleason scores from biopsy and after surgery and the tumor stages. Data are given in means, standard deviations, medians and ranges.

Abbreviations: PSMA = Prostate specific membrane antigen, PET = Positron emission tomography, MRI = Magnetic resonance imaging, PSA = Prostate specific antigen blood level.

### ^68^Ga-PSMA PET-CT and 3T MRI acquisition protocols

A standard ^68^Ge/^68^Ga generator (Eckert and Ziegler) was used for elution of ^68^Ga prior to labelling with PSMA-HBED-CC (ABX GmbH, Radeberg, Germany)^22,23,34^. After injection of 129.0 ± 26.2MBq of ^68^Ga-PSMA-HBED-CC, a low dose CT for attenuation correction (120 kVp, 30 mAs) and anatomical mapping was acquired within 89.0 ± 42.5 min immediately before the PET scan, using a Gemini TF 16 Astonish PET-CT scanner (Philips medical systems)^35^. All 130 patients underwent a non-contrast-enhanced prostate multiparametric MRI at our institution at 3T (Magentom Skyra, Siemens Healthcare, Erlangen, Germany). Standard prostate MRI acquisition protocol included high resolution T2 weighted high resolution turbo spin echo sequences (T2 HR TSE, 25 slices, thickness 3 mm, gap 3 mm, TR 4040 ms, TE 116 ms, resolution: 0.47 × 0.47 mm), including DWI sequences (DWI, 25 slices, thickness 3 mm, gap 3 mm, TR 4800 ms, TE 58 ms, resolution 1.4 × 1.4 mm and b-factors 0/160/1200 s/mm^2^) with generation of an ADC map from b 0, b 160 and b 1200 images of the DWI. Native T1 high resolution isotropic volume sequences (T1 TSC, 49 slices thickness 5 mm, gap 6 mm, TE 11 ms, TR 800 ms, resolution 0.63 × 0.63 mm). No endorectal coils were used throughout all exams.

### Image analysis

Visage 7.1 (Visage Imaging) was used as the standard software package. Low dose whole body CT sequences and ^68^Ga-PSMA PET sequences were automatically fused for the evaluation process. Since the acquisition of a MRI of the pelvis is limited to a localized area while ^68^Ga-PSMA PET-CT covered the whole body, the aortic bifurcation was set as the upper border for positive LN in ^68^Ga-PSMA PET-CT. Consensus reading was performed by two readers. All images were analysed independently in a blinded and random order.

### Assessment of lymph node metastasis in MRI

**Figure 5:**
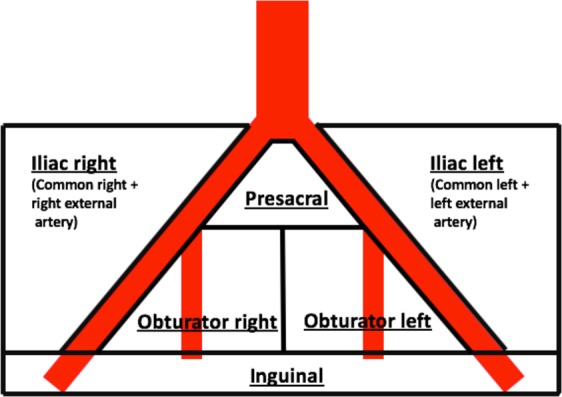
Template of Lymph node stations used in the region-based analysis. This figure shows the template of the lymph node (LN) stations examined in this study. The iliac left and right region included all LN around the common iliac and external iliac artery. The obturator left and right region included all LN around the internal iliac artery and its branches. The presacral region defined the area anterior of the sacrum without strict allocation to vessels. LN of the inguinal region were defined below the inguinal bands.

For LN examination in 3T MRI data sets, T1 TSE, T2 TSE and DWI sequences were used. T1 sequences were used in the first step, followed by high resolution T2 TSE and DWI to assess their additional value. The diameter of suspicious LN were measured in axial planes through manual delineation of a ROI, resulting in size, long-axis diameter (LAdm) and short axis diameter (SAdm). SAdm was defined as the rectangular line of the LAdm. A LN was defined as positive in MRI when the SAdm divided through LAdm exceeded the size ratio of 0.8 or if the LAdm was ≥10 mm, based on a previous study on LN detection in T1 sequences^29^. Signal intensity was no criteria for definition of a LN as metastatic. The size ratio is reported throughout the manuscript. All LN were characterized in six levels according to the adjacent anatomical structures dividing them into presacral, inguinal LN and alongside the large arterial vessels. LN alongside the common and external iliac arteries were characterized as the iliac right and iliac left region as superordinate levels and LN near the internal iliac and obturator artery were characterized as obturator right and obturator left region. Please refer to Fig. 5 for visualization of the defined regions.

### Measurement of lymph node metastasis in ^68^Ga-PSMA PET-CT

**Figure 6:**
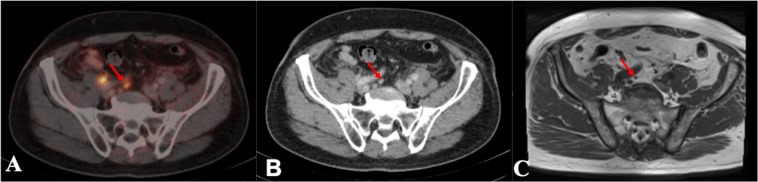
Example of a lymph node smaller than 10 mm in ^68^Gallium-Prostate specific membrane antigen PET-CT compared to T1 MRI. This figure shows a suspicious lymph node smaller than 10 mm diameter. The lymph node is visualized in corresponding axial plane slices using CT, ^68^Gallium-Prostate specific membrane antigen PET-CT and T1 MRI from left to right. The suspected lymph node is located at the right common iliac artery and is challenging to detect in T1 MRI. The lymph node is highlighted through red arrows. (**A**) Lymph node depicted in CT, (**B**) Lymph node depicted in ^68^Gallium-Prostate specific membrane antigen PET-CT, (**C**) Lymph node depicted in the T1 sequence in MRI.

**Figure 7:**
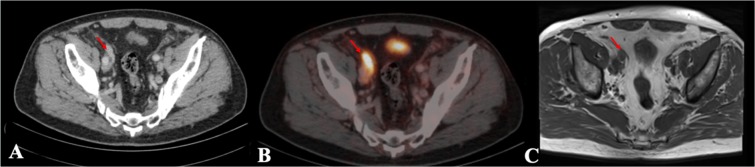
Example of a lymph node larger than 10 mm in ^68^Gallium-Prostate specific membrane antigen PET-CT compared to T1 MRI. This figure shows a suspicious lymph node larger than 10 mm diameter. The lymph node is visualized in corresponding axial plane slices using CT, ^68^Gallium-Prostate specific membrane antigen PET-CT and T1 MRI from left to right. The lymph node is located at the right external artery. The lymph node is highlighted through red arrows. (**A**) Lymph node depicted in CT, (**B**) Lymph node depicted in ^68^Gallium-Prostate specific membrane antigen PET-CT, (**C**) Lymph node depicted in the T1 sequence in MRI.

All ^68^Ga-PSMA avid LN were measured in the axial plane at maximum diameter using the CT sequence for delineation of the ROI after evaluation of tracer uptake in ^68^Ga-PSMA PET overlay at isocontour of 50%. LN were defined positive, when an abnormal focal tracer signal with a higher signal intensity than the surrounding background was detected in ^68^Ga-PSMA PET and a LN in CT could be allocated to the signal^14^. LN size evaluation did not affect the definition of positivity in ^68^Ga-PSMA PET-CT, which is comparable to a previously published study^14^. In addition, maximum standardized uptake values (SUVmax) were assessed. All visible LN on CT without tracer uptake were measured in LAdm and SAdm to be defined as true negatives. All LN depicted in PET-CT were divided into the 2 subgroups LN > 10 mm and LN ≤ 10 mm. MRI sensitivity was calculated for both size groups and for each anatomical region. Please refer to Fig. 6 for an example of a LN < 10 mm and Fig. 7 for a LN > 10 mm using PET-CT and MRI.

### Statistical analysis

^68^Ga-PSMA PET-CT was set as reference standard in this study.

Descriptive statistics were done using MedCalc Statistical Software version 17.6 (MedCalc Software bvba; http://www.medcalc.org; 2017) and R software (Version 3.5.0, Vienna, Austria, https://www.R-project.org, +lme4-package) was used for multi-level logistic regression. We used logistic regression in order to assess the diagnostic quality of MRI in contrast to our gold standard. The overall fit of a logistic regression model corresponds to the overall predictive accuracy and is as such related to comparable analysis techniques like ROC analysis. We utilized likelihood-ratio chi-square tests to calculate p-values within logistic regression. More specifically, we applied multi-level logistic regression to satisfy our hierarchical data structure (lymph nodes within patients) Multi-level regression adjusts for clusterings in data, i.e., for effects that some patients a conspicuous lymph node might be more likely to have more conspicuous lymph nodes^36^. Multi-level models include such effects and can even capture person-wise differences in diagnostic accuracy (called random slope models). In our analyses below, we tested for each predictive model whether such person-wise differences were statistically significant. The common properties of a diagnostical test (sensitivity, specificity, positive predictive value and negative predictive value) were calculated from logistic regression and confidence intervals (CI 95%) were given through logistic regression using the method proposed by Coughlin *et al*.^37^. When no confidence intervals could be calculated due to perfect agreement of MRI and PET-CT, it is highlighted in the tables. A p-value p < 0.05 was considered statistically significant.

## Supplementary information

Dataset 1
