# Supplementary material for: Accuracy of standard clinical 3T prostate MRI for pelvic lymph node staging: Comparison to68Ga-PSMA PET-CT
Source: Sci Rep. 2019 Jul 24;9:10727. doi: 10.1038/s41598-019-46386-3 (PMC6656738; doi:10.1038/s41598-019-46386-3)
Supplement: Supplementary file 1 — Dataset 1 [file 41598_2019_46386_MOESM1_ESM.pdf]

# Accuracy of standard clinical 3T prostate MRI

# for pelvic lymph node staging:

# *Comparison to $^{68}$Ga-PSMA PET-CT*

Sebastian Meißner[1]; Jan-Carlo Janssen[1]; Vikas Prasad[2], MD; Gerd Diederichs[1], MD; Bernd Hamm[1] MD, PhD, Winfried Brenner[2], MD, PhD; Marcus R. Makowski[1], MD, PhD.

[1] Department of Radiology, Charity, Charitéplatz 1, 10117 Berlin, Germany.

[2] Department of Nuclear Medicine, Charité, Charitéplatz 1, 10117 Berlin, Germany.

Manuscript category: Original article

Sebastian Meißner

Department of Radiology,

Charité Universitätsmedizin Berlin, Berlin, Germany.

sebastian.meissner@charite.de

phone: +49 30 450 645373

**Supplementary data**

***Detection of lymph node metastasis in MRI dependent on lymph node size***

To assess a possible impact of LN size in MRI detection, 2 groups were defined using the LAdm cut-off at 10mm in CT. For all LN, that were observed in PET-CT and MRI, the LN area was marginally larger in CT than in MRI. On average, the LN area in the MRI comprised 95.5% of the CT area and did not change significantly with longer delay between both scans ($\chi^2(2)=5.5$, $p=0.06$). patients with the shortest delay presented a congruence of 96.6 while patients with the longest delays had 93.8%. LN with a LAdm ≤10mm in CT were detected with a sensitivity, specificity, PPV and NPV of 69.4% (CI 54.5%-81.1%), 99.5% (CI 99.0%-99.7%), 74.0% (CI 43.3%-91.4%) and 99.5% (CI 98.1%-99.9%) in MRI. They had a mean area of 0.4±0.3cm$^2$ (range 0.1–1.6cm$^2$) and a mean size ratio of 0.9±0.4 (range 0.3-3.5) in MRI. False negative LN had a mean area, size ratio and SUVmax of 0.4±0.3cm$^2$ (range 0.1–1.8cm$^2$), 0.7±0.1 (range 0.5–1.0)and 5.8±3.8 (range 1.3-18.9) in $^{68}$Ga-PSMA-PET-CT. False positive LN had a mean area of 1.1±0.7cm$^2$ (range 0.2-3.0cm$^2$) and a size ratio of 0.7±0.2 (range 0.4-1.0) in MRI. LN with LAdm >10mm in CT were detected with a sensitivity, specificity, PPV and NPV of 92.0% (CI 83.0%-96.5%), 95.6% (CI 90.2%-98.1%), 72.9% (CI 48.8%-88.4%) and 99.7% (CI 99.2%-99.9%) in MRI. They had a mean area of 1.4±1.5cm$^2$ (range 0.1-9.1cm$^2$) and a size ratio of 0.8±0.5 (range 0.3–3.4). False negative LN metastases had mean area, size ratio and SUVmax of 0.7±0.4cm$^2$ (range 0.4–1.6cm$^2$), 0.6±0.1 (range 0.4–0.8) and 7.0±3.8 (range 0.7–10.4) in $^{68}$Ga-PSMA-PET-CT. False positive LN had a mean area of 1.3±0.7cm$^2$ (range 0.3–3.0cm$^2$) and a size ratio of 0.9+0.1 (range 0.5-0.9) in MRI.

Overall, sensitivity in MRI did not differ significantly for both size groups ($x^2(1)=0.62$, $p=0.43$) although sensitivity in the ≤10mm group (69%) tended to be lower than in the >10mm group (92%). More details are provided in supplementary table 1.

| T1+T2 | N | LN Size | Sensitivity + 95 % CI | Specificity + 95 % CI | PPV + 95 % CI | NPV + 95 % CI | p – value (accuracy) |
|---|---|---|---|---|---|---|---|
| | | | | | | | |
| **LN** | **146 / 187** | **All** | **81.6 %<br>(71.1 % - 88.9 %)** | **98.6 %<br>(97.6 5 – 99.2 %)** | **73.5 %<br>(52.1 % - 87.6 %)** | **99.5 %<br>(98.8 % - 99.8 %)** | |
| | **60 / 92** | **≤ 10mm** | 69.4 %<br>(54.5 % - 81.1 %) | 99.5 %<br>(99.0 % - 99.7 %) | 74.0 %<br>(43.3 % - 91.4 %) | 99.5 %<br>(98.1 % - 99.9 %) | p = 0.301 |
| | **86 / 95** | **> 10mm** | 92.0 %<br>(83.0 % - 96.5 %) | 95.6 %<br>(90.2 % - 98.1 %) | 72.9 %<br>(48.8 % - 88.4 %) | 99.7 %<br>(99.2 % - 99.9 %) | |
| **Iliac left** | **7 / 13** | **≤ 10mm** | 63.2 %<br>(33.2 % - 85.6 %) | 99.3 %<br>(97.6 % - 99.8 %) | 49.3 %<br>(7.0 % - 92.6 %) | 99.7 %<br>(96.1 % - 100 %) | p = 0.088 |
| | **16 / 18** | **> 10mm** | 92.0 %<br>(68.7 % - 99.0 %) | 89.0 %<br>(60.1 % - 98.0 %) | 53.0 %<br>(16.4 % - 87.0 %) | 99.0 %<br>(94.6 % - 100 % ) | |
| **Iliac right** | **5 / 15** | **≤ 10mm** | 35.4 %<br>(13.4 % - 65.8 %) | 99.1 %<br>(96.8 % - 99.7 %) | 62.5 %<br>(10.1 % - 96.1 %) | 99.2 %<br>(89.3 % - 99.9 %) | p = 0.715 |
| | **25 / 32** | **> 10mm** | 80.0 %<br>(59.0 % -92.0 %) | 83.0 %<br>(63.2 % - 93.0 %) | 77.0 %<br>(36.7 % - 95.0 %) | 96.0 %<br>(81.4 % - 99.0 %) | |
| **Obturator left** | **14 / 19** | **≤ 10mm** | 72.1 %<br>(42.9 % - 89.9 %) | 98.2 %<br>(94.0 % - 99.4 %) | 61.5 %<br>(12.0 % - 94.9 %) | 98.6 %<br>(86.0 % - 99.9 %) | p = 0.193 |
| | **14 / 14** | **> 10mm** | 100 %<br>(0 % - 100 %) | 100 %<br>(0 % - 100 %) | 100 %<br>(0 % - 100 %) | 100 %<br>(0 % - 100 %) | |
| **Obturator right** | **23 / 31** | **≤ 10mm** | 78.6 %<br>(57.9 % - 90.8 %) | 97.3 %<br>(93.0 % - 99.0 %) | 86.6 %<br>(36.5 % - 98.6%) | 96.9 %<br>(73.5 % - 99.7 %) | p = 0.334 |
| | **21 / 21** | **> 10mm** | 100 %<br>(0 % - 100 %) | 88.0 %<br>(0 % - 100 %) | 96.0 %<br>(57.1 % - 100 %) | 100 %<br>(0 % - 100 %) | |
| **Presacral** | **8 / 11** | **≤ 10mm** | 75.4<br>(40.2 % - 93.3 %) | 100 %<br>(0 % - 100 %) | 99.7 %<br>(0 % - 100 %) | 95.4 %<br>(0 % - 100 %) | p = 0.334 |
| | **5 / 5** | **> 10mm** | 100 %<br>a | 100 %<br>a | 100 %<br>a | 100 %<br>a | |
| **Inguinal** | **3 / 3** | **≤ 10mm** | 100 %<br>(0 % - 100 %) | 100 %<br>(0 % - 100 %) | 100 %<br>(0 % - 100 %) | 100 %<br>(0 % - 100 %) | p = 1.0 |
| | **5 / 5** | **> 10mm** | 100 %<br>(0 % - 100 %) | 97.0 %<br>(0 % - 100 %) | 21.0 %<br>(3.4 % - 67.0 %) | 100 %<br>(0 % - 100 %) | |
| a = CI could not be calculated as PET-CT and MRI agreed perfectly in this case. | | | | | | | |

**Supplementary table 1. Size-based analysis of the detection rates using combined T1+T2+DWI sequence MRI evaluation**

This table summarizes the size-based analysis of the lymph node metastases in MRI using T1 combined with T2 and DWI sequence evaluation. All $^{68}$Gallium-Prostate membrane antigen PET-CT positive metastases were categorized in two subgroups with a long-axis diameter ≤10mm and >10mm measured in axial plane. The columns present the numbers of seen LN compared to PET-CT, sensitivities, specificities, positive and negative predictive value including 95% confidence interval and the p-values of differences in the accuracy of detection. The rows show the overall values of all found metastases ≤10mm and >10mm and fielded in the six defined anatomical regions iliac left and right, obturator left and right, presacral and inguinal region. A p-value $p<0.05$ was considered statistically significant.

When no confidence intervals could be calculated due to perfect agreement of MRI and PET-CT, it is highlighted in the table with an “a”.

Abbreviations: T2 = T2 weighted sequence, DWI = diffusion weighted imaging sequence, LN = Lymph nodes, N = number of positive LN in MRI versus PET-CT, PPV = positive predictive value, NPV = Negative predictive value, CI = 95% Confidence interval

***Detection of lymph node metastasis in MRI dependent on size and anatomical region***

Furthermore, the two size groups were examined based on the anatomical regions. No significant differences were seen throughout all anatomical regions. Although a tendency was seen for the iliac left region ($x^2(1)=2.9$, $p=0.08$). Further details are provided in supplementary figure 1.

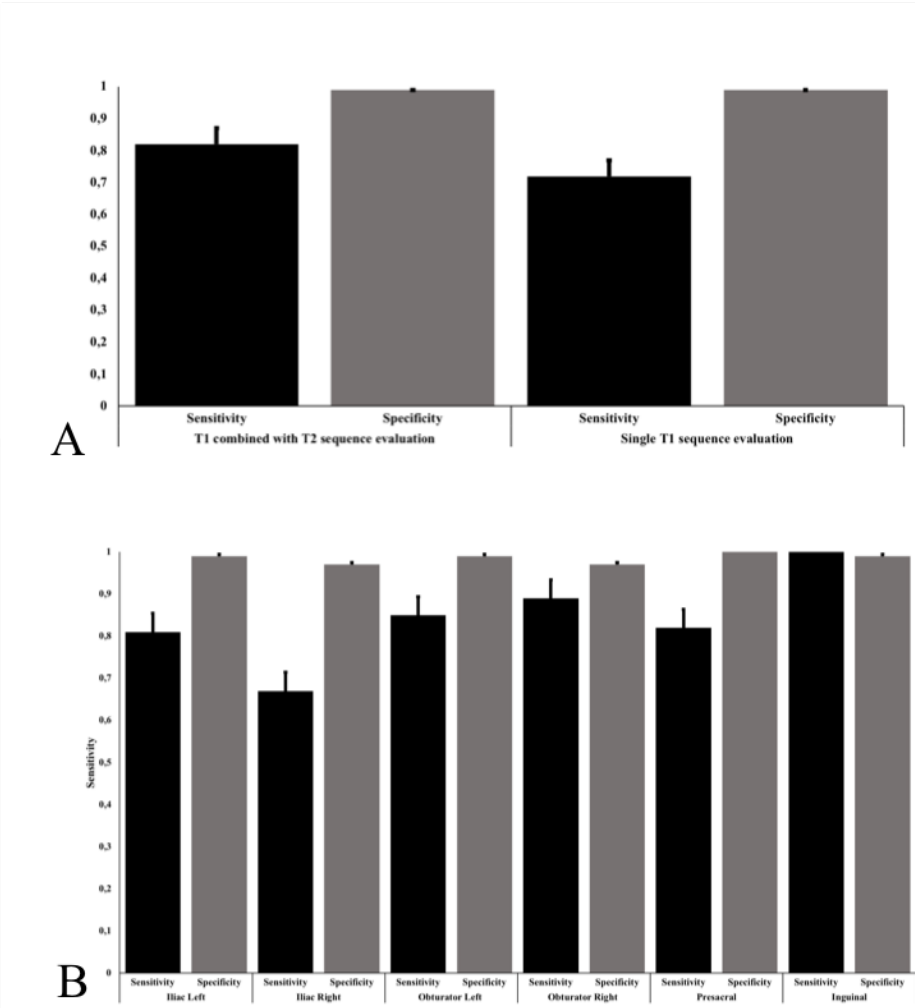


**Supplementary figure 1: Visualisation of the detection rates of lymph node metastases smaller and larger than 10mm in MRI**

A: This bar chart presents the accuracy of the detection for lymph node (LN) metastases smaller 10mm versus larger than 10mm showing the overall sensitivities and specificities. No significant (p>0.05) difference in the accuracy was seen. B: This bar chart presents the accuracy of the detection for LN metastases smaller 10mm versus larger than 10mm on a region-based

analysis in the six defined anatomical regions iliac left and right, obturator left and right, presacral and inguinal region. No significance ($p>0.05$) was seen throughout all anatomical regions.

Abbreviations: LN = Lymph nodes

***Impact of the additional T2 and DWI sequences in the detection of lymph node metastasis dependent on lymph node size***

LN with a LAdm ≤10mm in CT were detected in with a sensitivity, specificity, PPV and NPV of 73.7% (CI 56.9%-85.6%), 99.7% (CI 99.4%-99.9%), 77.8% (CI 45.1%-93.7%) and 99.6% (CI 99.3%-99.9%) in single T1 sequence evaluation. They had a mean area of 0.4±0.3$cm^2$ (range 0.1–1.6$cm^2$) and a mean size ratio of 0.9±0.1 (range 0.3-1.0) was in single T1 evaluation. False negative LN had a mean area, size ratio and SUVmax of 0.4±0.3$cm^2$ (range 0.1–1.8$cm^2$), 0.7±0.2 (range 0.5–1.5) and 5.8±3.8 (range 1.3-18.9) in $^{68}$Ga-PSMA-PET-CT. the 2 false positive LN had a mean area of 0.9±0.7$cm^2$ (range 0.2-3.0$cm^2$) and a mean size ratio of 0.9 in single T1 evaluation. LN with LAdm >10mm in CT were detected with a sensitivity, specificity, PPV and NPV of 70.0% (CI 52.6%-83.1%), 97.1% (CI 94.1%-98.6%), 69.8% (CI 43.0%-87.6%) and 99.1% (CI 97.2%-99.7%) in single T1 evaluation. They had a mean area of 1.3±1.4$cm^2$ (range 0.1-9.1$cm^2$) and a size ratio of 0.7±0.2 (range 0.3–1.0). False negative LN metastases had mean area, size ratio and SUVmax of 0.8±0.3$cm^2$ (range 0.4–1.6$cm^2$), 0.6±0.1 (range 0.4–0.8) and 6.8±3.6 (range 0.7–12.8) in $^{68}$Ga-PSMA-PET-CT. False positive LN had a mean area of 0.9±0.3$cm^2$ (range 0.6–1.3$cm^2$) and a size ratio of 0.9±0.2 (range 0.5-1.0).

Overall sensitivity in single T1 sequence evaluation did not differ significantly for both size groups since the sensitivity in the ≤10mm group (73.7%) was only marginally higher than in the >10mm group (70%) and most of the CI overlapped. The same non-significant effect was

seen in the assessment of sensitivity differences based on size and anatomical regions. More details are provided in supplementary table 1.

| Single T1 | N | LN Size | Sensitivity + 95 % CI | Specificity + 95 % CI | PPV + 95 % CI | NPV + 95 % CI |
|---|---|---|---|---|---|---|
| | | | | | | |
| **LN** | **130 / 187** | **All** | **81.6 %<br>(71.1 % - 88.9 %)** | **98.6 %<br>(97.6 5 – 99.2 %)** | **73.5 %<br>(52.1 % - 87.6 %)** | **99.5 %<br>(98.8 % - 99.8 %)** |
| | | | | | | |
| | **50/ 92** | **≤ 10mm** | **73.7 %<br>(56.9 % - 85.6 %)** | **99.7 %<br>(99.4 % - 99.9 %)** | **77.8 %<br>(45.1 % - 93.7 %)** | **99.6 %<br>(99.3 % - 99.9 %)** |
| | **80 / 95** | **> 10mm** | **70.0 %<br>(52.6 % - 83.1 %)** | **97.1 %<br>(94.1 % – 98.6 %)** | **69.8 %<br>(43.0 % - 87.6 %)** | **99.1 %<br>(97.2 % - 99.7 %)** |
| | | | | | | |
| **Iliac left** | **5 / 13** | **≤ 10mm** | 75.0 %<br>(43.6 % - 92.0 %) | 100 %<br>(81.7 % - 100 %) | 42.0 %<br>(3.9 % - 93.0 %) | 100 %<br>(98.0 % - 100 %) |
| | **16 /18** | **> 10mm** | 72.0 %<br>(41.6 % - 90.0 %) | 95.0 %<br>(82.7 % - 98.0 %) | 54.0 %<br>(12.9 % - 90.0 %) | 98.0 %<br>(88.4 % - 100 %) |
| **Iliac right** | **4 / 15** | **≤ 10mm** | 51.0 %<br>(21.1 % - 80.0 %) | 100 %<br>(98.8 % - 100 %) | 90 %<br>(10.2 % - 100 %) | 100 %<br>(81.7 – 100 %) |
| | **24 / 32** | **> 10mm** | 71.0 %<br>(46.1 % - 87.0 %) | 87.0 %<br>(69.4 % - 95.0 %) | 72.0 %<br>(28.2 % – 95.0 %) | 95.0 %<br>(74.5 % - 99.0 %) |
| **Obturator left** | **13 / 19** | **≤ 10mm** | 80.0 %<br>(48.5 5 – 94.0 %) | 100 %<br>(97.8 % - 100 %) | 90.0 %<br>(15.9 % - 100 %) | 100 %<br>(82.0 % - 100 %) |
| | **11 / 14** | **> 10mm** | 69.0 %<br>(29.3 % - 93.0 %) | 100 %<br>(0 % - 100 %) | 100 %<br>(0 % - 100 %) | 45.0 %<br>(0 % - 100 %) |
| **Obturator right** | **17 / 31** | **≤ 10mm** | 74.0 %<br>(51.2 % - 89.0 %) | 98.0 %<br>(95.0 % - 99.0 %) | 73.0 %<br>(16.2 % - 97.0 %) | 98.0 %<br>(78.4 % - 100 %) |
| | **19 / 21** | **> 10mm** | 56.0 %<br>(28.8 % - 80.0 %) | 93.0 %<br>(80.4 % - 98.0 %) | 95.0 %<br>(40.1 % - 100 %) | 36.0 %<br>(1.9 % - 94.0 %) |
| **Presacral** | **8 / 11** | **≤ 10mm** | 77.0 %<br>(41.1 % - 94.0 %) | 100 %<br>(0 % - 100 %) | 100 %<br>(0 % - 100 %) | 98.0 %<br>(0 %- 100 %) |
| | **5 / 5** | **> 10mm** | 100 %<br>a | 100 %<br>a | 100 %<br>a | 100 %<br>a |
| **Inguinal** | **3 / 3** | **≤ 10mm** | 100 %<br>(0 % - 100 %) | 100 %<br>(0 % - 100 %) | 100 %<br>(0 % - 100 %) | 100 %<br>(0 % - 100 %) |
| | **5 / 5** | **> 10mm** | 100 %<br>(0 % - 100 %) | 98.0 %<br>(0 % - 100 %) | 20.0 %<br>(2.5 % – 71.0 %) | 100 %<br>(0 % - 100 %) |
| a = CI could not be calculated as PET-CT and MRI agreed perfectly in this case. | | | | | | |

**Supplementary table 2. Size-based analysis of the detection rates using single T1 sequence MRI evaluation**

This table summarizes the size-based analysis of the lymph node metastases in MRI using single T1 sequence evaluation. All $^{68}$Gallium-Prostate membrane antigen PET-CT positive metastases were categorized in two subgroups with a long-axis diameter ≤10mm and >10mm measured in axial plane. The columns present the numbers of seen LN compared to PET-CT, sensitivities, specificities, positive and negative predictive value including 95% confidence intervals. The rows show the overall values of all found metastases ≤10mm and >10mm and fielded in the six defined anatomical regions iliac left and right, obturator left and right, presacral and inguinal region.

When no confidence intervals could be calculated due to perfect agreement of MRI and PET-CT, it is highlighted in the table with an “a”.

Abbreviations: LN = Lymph nodes, N = number of positive LN in MRI versus PET-CT, PPV = positive predictive value, NPV = Negative predictive value, CI = 95% Confidence interval

## References


1 Prasad V, Steffen IG, Diederichs G, Makowski MR, Wust P, Brenner W (2016) Biodistribution of [(68)Ga]PSMA-HBED-CC in Patients with Prostate Cancer: Characterization of Uptake in Normal Organs and Tumour Lesions. Mol Imaging Biol 18:428-436

2 Dietlein M, Kobe C, Kuhnert G et al (2015) Comparison of [(18)F]DCFPyL and [(68)Ga]Ga-PSMA-HBED-CC for PSMA-PET Imaging in Patients with Relapsed Prostate Cancer. Mol Imaging Biol 17:575-584

3 Afshar-Oromieh A, Malcher A, Eder M et al (2013) PET imaging with a [68Ga]gallium-labelled PSMA ligand for the diagnosis of prostate cancer: biodistribution in humans and first evaluation of tumour lesions. Eur J Nucl Med Mol Imaging 40:486-495

4 Surti S, Kuhn A, Werner ME, Perkins AE, Kolthammer J, Karp JS (2007) Performance of Philips Gemini TF PET/CT scanner with special consideration for its time-of-flight imaging capabilities. J Nucl Med 48:471-480
